# Supplementary material for: T2*-Mapping of Knee Cartilage in Response to Mechanical Loading in Alpine Skiing: A Feasibility Study
Source: Diagnostics (Basel). 2022 Jun 4;12(6):1391. doi: 10.3390/diagnostics12061391 (PMC9222057; doi:10.3390/diagnostics12061391)
Supplement: Supplementary file 1 [file diagnostics-12-01391-s001.zip › diagnostics-1744737-supplementary.pdf]

**Supplementary Materials:**

## Sublementary files

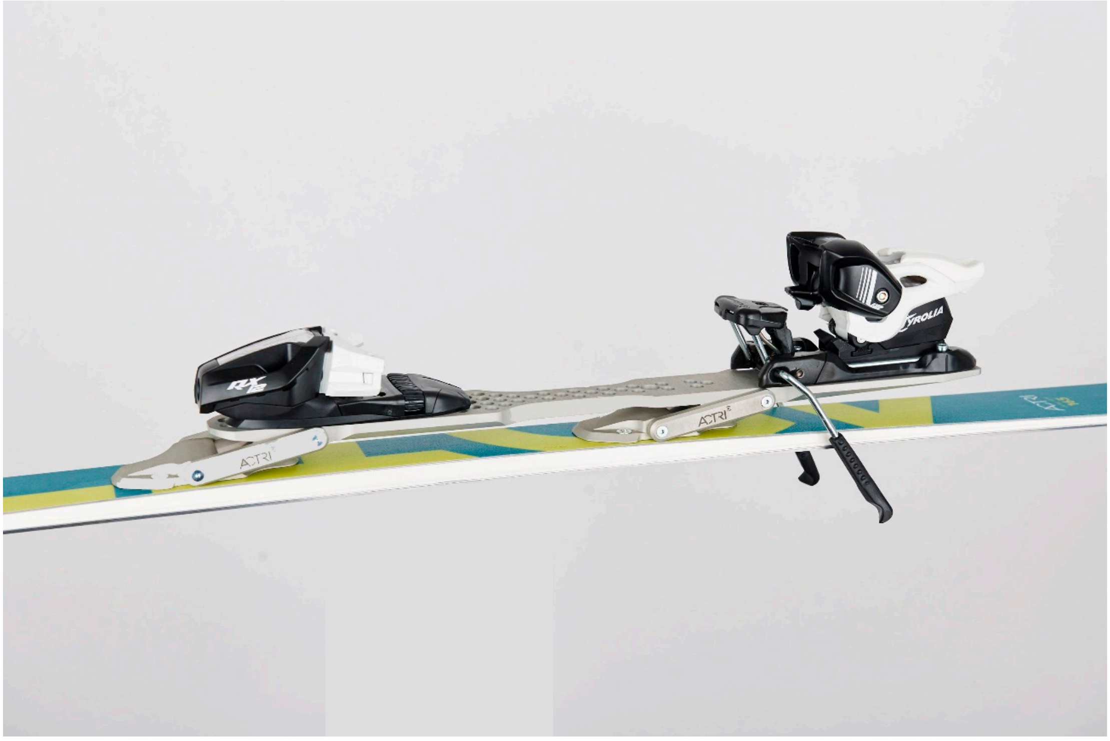

**Figure S1:** Ski with Suspended Ski Binding System ACTRI F. The damping plate consists of duralumin with pressed-in plain bearing sleeves and inserted leaf spring made of austenitic stainless chromium-nickel steel.

**Table S1:** Mean chondral Ht (mm) in FTJ and retropatellar.

| joint | side | subject | baseline 1 | fu 1-1 | fu 1-2 | recovery | baseline 2 | fu 2-1 | fu 2-2 | mean SD |
|---|---|---|---|---|---|---|---|---|---|---|
| **FTJ lateral femoral** | **right** | **1** | 2.53 | 2.39 | 2.48 | 2.68 | 2.41 | 2.03 | 2.28 | **0.22** |
| | | **2** | 3.04 | 2.75 | 3.41 | 2.85 | 3.15 | 3.00 | 2.96 | **0.24** |
| | | **3** | 2.40 | 2.25 | 2.34 | 2.99 | 2.54 | 2.39 | 2.48 | **0.27** |
| | | **4** | 3.24 | 2.71 | 3.11 | 3.35 | 3.29 | 2.91 | 3.14 | **0.30** |
| | **left** | **1** | 2.48 | 2.29 | 2.21 | 2.28 | 2.26 | 2.14 | 2.19 | **0.16** |
| | | **2** | 3.31 | 2.80 | 2.93 | 2.90 | 2.96 | 2.98 | 2.99 | **0.38** |
| | | **3** | 2.36 | 2.14 | 2.23 | 2.29 | 2.40 | 2.20 | 2.21 | **0.16** |
| | | **4** | 2.81 | 2.94 | 2.78 | 2.49 | 2.76 | 2.50 | 2.66 | **0.21** |
| **FTJ lateral tibial** | **right** | **1** | 3.25 | 3.26 | 3.16 | 3.23 | 3.08 | 2.73 | 3.13 | **0.24** |
| | | **2** | 2.76 | 2.83 | 3.13 | 2.85 | 2.65 | 2.74 | 2.88 | **0.24** |
| | | **3** | 2.49 | 2.29 | 2.30 | 2.81 | 2.55 | 2.16 | 2.35 | **0.24** |
| | | **4** | 3.23 | 2.79 | 2.91 | 3.29 | 3.21 | 2.99 | 3.18 | **0.26** |
| | **left** | **1** | 3.09 | 2.69 | 2.49 | 2.63 | 2.63 | 2.51 | 2.58 | **0.25** |
| | | **2** | 2.98 | 2.78 | 2.89 | 2.99 | 2.83 | 2.84 | 2.84 | **0.17** |
| | | **3** | 2.33 | 2.00 | 2.18 | 2.39 | 2.40 | 2.36 | 2.29 | **0.18** |
| | | **4** | 3.30 | 3.06 | 3.01 | 2.84 | 3.19 | 3.00 | 3.24 | **0.25** |
| **FTJ medial femoral** | **right** | **1** | 2.64 | 2.36 | 2.51 | 2.60 | 2.55 | 2.38 | 2.68 | **0.21** |
| | | **2** | 2.48 | 2.41 | 2.50 | 2.45 | 2.63 | 2.71 | 2.65 | **0.15** |
| | | **3** | 1.71 | 1.71 | 1.58 | 1.91 | 1.86 | 1.84 | 1.66 | **0.16** |
| | | **4** | 2.59 | 2.66 | 2.43 | 2.76 | 2.33 | 2.69 | 2.66 | **0.21** |
| | **left** | **1** | 2.59 | 2.85 | 2.76 | 2.79 | 2.45 | 2.74 | 2.64 | **0.16** |
| | | **2** | 2.66 | 2.98 | 2.44 | 2.65 | 2.59 | 2.63 | 2.33 | **0.27** |
| | | **3** | 1.99 | 1.65 | 1.65 | 1.90 | 1.73 | 1.78 | 1.74 | **0.18** |
| | | **4** | 2.90 | 2.58 | 2.70 | 2.56 | 2.55 | 2.61 | 2.70 | **0.20** |
| **FTJ medial tibial** | **right** | **1** | 1.70 | 1.98 | 1.93 | 1.98 | 1.90 | 1.98 | 2.05 | **0.18** |
| | | **2** | 1.75 | 1.81 | 2.04 | 1.89 | 2.03 | 1.79 | 1.99 | **0.20** |
| | | **3** | 1.68 | 1.81 | 1.81 | 1.65 | 1.79 | 1.66 | 1.53 | **0.19** |
| | | **4** | 1.89 | 1.94 | 1.91 | 1.76 | 1.98 | 1.91 | 1.84 | **0.17** |
| | **left** | **1** | 1.88 | 2.15 | 2.06 | 1.96 | 1.98 | 1.95 | 1.94 | **0.16** |
| | | **2** | 2.25 | 2.35 | 1.90 | 2.10 | 2.15 | 2.13 | 1.98 | **0.26** |
| | | **3** | 1.91 | 1.84 | 1.68 | 1.58 | 1.84 | 1.71 | 1.76 | **0.16** |
| | | **4** | 2.01 | 1.89 | 1.83 | 1.99 | 2.00 | 1.71 | 1.75 | **0.18** |
| **FPJ retro-pattelar** | **right** | **1** | 3.56 | 3.23 | 3.71 | 3.70 | 3.63 | 3.40 | 3.41 | **0.27** |
| | | **2** | 3.05 | 2.91 | 3.16 | 2.63 | 2.78 | 2.55 | 2.80 | **0.45** |
| | | **3** | 2.44 | 2.10 | 2.11 | 2.14 | 2.50 | 2.51 | 2.31 | **0.25** |
| | | **4** | 2.40 | 2.18 | 2.38 | 2.54 | 2.49 | 2.28 | 2.28 | **0.20** |
| | **left** | **1** | 3.49 | 3.65 | 3.58 | 3.34 | 3.71 | 3.34 | 3.54 | **0.31** |
| | | **2** | 2.79 | 2.78 | 2.54 | 2.60 | 2.91 | 3.14 | 2.73 | **0.29** |
| | | **3** | 2.40 | 2.09 | 2.38 | 2.21 | 2.05 | 2.25 | 1.99 | **0.27** |
| | | **4** | 3.43 | 2.83 | 3.14 | 3.33 | 3.11 | 3.11 | 3.20 | **0.33** |

**Table S2:** Mean Segmental Intrachondral T2*-time (ms) with SD in FTJ and retropatellar.

| joint | side | subject | baseline 1 | fu 1-1 | fu 1-2 | recovery | baseline 2 | fu 2-1 | fu 2-2 | mean SD |
|---|---|---|---|---|---|---|---|---|---|---|
| **FTJ lateral femoral** | **right** | **1** | 30.7 | 29.0 | 29.6 | 31.7 | 30.3 | 30.9 | 32.1 | **1.85** |
| | | **2** | 31.9 | 30.6 | 31.3 | 31.1 | 31.5 | 31.6 | 32.0 | **1.93** |
| | | **3** | 26.8 | 26.2 | 24.6 | 25.2 | 26.1 | 27.9 | 24.2 | **2.23** |
| | | **4** | 25.5 | 27.8 | 26.2 | 30.0 | 26.7 | 26.0 | 26.6 | **2.32** |
| | **left** | **1** | 29.8 | 31.6 | 32.1 | 33.3 | 32.0 | 32.8 | 34.1 | **2.13** |
| | | **2** | 31.1 | 29.7 | 30.2 | 31.5 | 30.8 | 30.8 | 30.8 | **1.88** |
| | | **3** | 25.3 | 25.7 | 23.9 | 25.8 | 25.6 | 25.4 | 26.4 | **1.52** |
| | | **4** | 26.2 | 26.2 | 28.2 | 29.8 | 26.5 | 27.6 | 25.9 | **2.42** |
| **FTJ lateral tibial** | **right** | **1** | 18.9 | 19.7 | 20.1 | 20.3 | 19.3 | 22.1 | 20.8 | **1.58** |
| | | **2** | 19.2 | 18.7 | 18.7 | 19.8 | 18.7 | 19.3 | 19.4 | **1.32** |
| | | **3** | 18.3 | 17.5 | 17.5 | 15.6 | 17.3 | 18.5 | 16.4 | **1.40** |
| | | **4** | 19.2 | 21.4 | 20.4 | 19.7 | 20.4 | 20.5 | 20.0 | **1.48** |
| | **left** | **1** | 22.2 | 23.4 | 24.4 | 24.6 | 22.4 | 24.1 | 23.1 | **1.81** |
| | | **2** | 19.5 | 20.1 | 18.7 | 20.0 | 20.4 | 19.4 | 18.0 | **1.48** |
| | | **3** | 17.8 | 18.6 | 17.4 | 17.7 | 17.4 | 17.6 | 17.2 | **1.16** |
| | | **4** | 20.4 | 20.2 | 21.5 | 23.2 | 21.4 | 20.3 | 19.9 | **1.78** |
| **FTJ medial femoral** | **right** | **1** | 28.8 | 26.4 | 26.5 | 28.0 | 26.4 | 26.6 | 27.8 | **2.10** |
| | | **2** | 29.9 | 28.3 | 25.1 | 27.7 | 27.9 | 28.9 | 26.6 | **2.42** |
| | | **3** | 22.5 | 22.7 | 22.2 | 22.1 | 23.8 | 23.5 | 23.1 | **1.56** |
| | | **4** | 27.1 | 27.3 | 26.5 | 28.1 | 26.6 | 26.8 | 25.9 | **1.70** |
| | **left** | **1** | 28.0 | 27.2 | 26.9 | 28.2 | 27.2 | 27.6 | 27.1 | **1.47** |
| | | **2** | 30.2 | 30.3 | 28.5 | 31.5 | 30.3 | 29.0 | 27.4 | **3.03** |
| | | **3** | 22.6 | 24.2 | 23.2 | 23.3 | 23.5 | 23.4 | 22.3 | **1.20** |
| | | **4** | 24.0 | 24.7 | 24.3 | 28.1 | 25.3 | 24.9 | 23.4 | **2.13** |
| **FTJ medial tibial** | **right** | **1** | 24.1 | 23.5 | 21.7 | 23.7 | 22.9 | 21.7 | 22.3 | **1.74** |
| | | **2** | 27.5 | 25.0 | 25.0 | 24.2 | 25.2 | 27.3 | 22.8 | **2.63** |
| | | **3** | 22.6 | 21.8 | 20.3 | 22.6 | 22.8 | 22.1 | 21.4 | **1.64** |
| | | **4** | 22.7 | 21.4 | 22.4 | 23.3 | 23.0 | 23.1 | 23.1 | **1.42** |
| | **left** | **1** | 21.7 | 19.8 | 20.8 | 22.4 | 22.8 | 21.6 | 22.0 | **1.66** |
| | | **2** | 24.8 | 21.4 | 25.4 | 25.6 | 24.5 | 24.1 | 23.3 | **3.21** |
| | | **3** | 20.8 | 21.6 | 20.8 | 22.6 | 22.0 | 21.7 | 20.5 | **1.38** |
| | | **4** | 19.2 | 20.6 | 19.9 | 20.1 | 20.8 | 19.7 | 21.4 | **1.52** |
| **FPJ retro-pattelar** | **right** | **1** | 29.9 | 28.2 | 29.2 | 33.0 | 30.7 | 30.1 | 29.7 | **2.20** |
| | | **2** | 29.7 | 28.9 | 29.9 | 30.3 | 28.5 | 28.1 | 29.0 | **2.68** |
| | | **3** | 27.1 | 24.3 | 25.3 | 26.0 | 25.6 | 26.2 | 25.8 | **1.40** |
| | | **4** | 21.0 | 24.1 | 22.6 | 22.2 | 23.1 | 21.9 | 23.6 | **1.61** |
| | **left** | **1** | 30.8 | 29.7 | 28.2 | 31.6 | 30.2 | 29.0 | 29.6 | **2.34** |
| | | **2** | 27.9 | 28.3 | 27.6 | 30.4 | 29.7 | 28.5 | 29.3 | **2.09** |
| | | **3** | 26.6 | 27.0 | 25.9 | 26.8 | 25.8 | 26.5 | 25.2 | **1.57** |
| | | **4** | 20.0 | 21.4 | 21.1 | 21.0 | 20.8 | 20.7 | 21.0 | **1.21** |
